# Flow Cytometric Clinical Immunomonitoring Using Peptide–MHC Class II Tetramers: Optimization of Methods and Protocol Development

**DOI:** 10.3389/fimmu.2018.00008

**Published:** 2018-01-22

**Authors:** Diahann T. S. L. Jansen, Nishta Ramnoruth, Khai L. Loh, Jamie Rossjohn, Hugh H. Reid, Hendrik J. Nel, Ranjeny Thomas

**Affiliations:** ^1^University of Queensland Diamantina Institute, Translational Research Institute, Princess Alexandra Hospital, Brisbane, QLD, Australia; ^2^Infection and Immunity Program and The Department of Biochemistry and Molecular Biology, Biomedicine Discovery Institute, Monash University, Clayton, VIC, Australia; ^3^ARC Centre of Excellence in Advanced Molecular Imaging, Monash University, Clayton, VIC, Australia; ^4^School of Medicine, Institute of Infection and Immunity, Cardiff University, Cardiff, United Kingdom

**Keywords:** antigen-specific T cells, tetramers, tetramerization, protocol standardization, flow cytometry

## Abstract

With the advent of novel strategies to induce tolerance in autoimmune and autoimmune-like conditions, clinical trials of antigen-specific tolerizing immunotherapy have become a reality. Besides safety, it will be essential to gather mechanistic data on responding CD4^+^ T cells to assess the effects of various immunomodulatory approaches in early-phase trials. Peptide–MHC class II (pMHCII) multimers are an ideal tool for monitoring antigen-specific CD4^+^ T cell responses in unmanipulated cells directly *ex vivo*. Various protocols have been published but there are reagent and assay limitations across laboratories that could hinder their global application to immune monitoring. In this methodological analysis, we compare protocols and test available reagents to identify sources of variability and to determine the limitations of the tetramer binding assay. We describe a robust pMHCII flow cytometry-based assay to quantify and phenotype antigen-specific CD4^+^ T cells directly *ex vivo* from frozen peripheral blood mononuclear cell samples, which we suggest should be tested across various laboratories to standardize immune-monitoring results.

## Introduction

Autoimmune diseases result from chronic T cell and B cell autoimmune responses leading to tissue inflammation and damage. Helper CD4^+^ T cells play a central role in autoimmune responses because they orchestrate the function of other immune cells including cytotoxic T cells and B cells. Current treatment strategies for autoimmune diseases target inflammation, the whole immune system, or the total T cell or B cell populations, modulating the immune response in antigen non-specific approaches. Antigen-specific tolerizing immunotherapy specifically targets the autoimmune response leaving the rest of the immune system unimpaired. Several different approaches to induce antigen-specific tolerance have been developed over recent years, and the first clinical trials are completed and are in progress (1, 2). These immunomodulatory approaches require the development of tools to assess the efficacy of antigen-specific tolerizing immunotherapy. To monitor antigen-specific CD4^+^ T cell responses, antigen-specific CD4^+^ T cells can be restimulated with peptide *ex vivo* for analysis of proliferation and cytokine production. However, responses to autoantigenic peptides in human PB are typically much lower and more variable than in mouse models of autoimmune disease. Autoantibody titers provide an indirect measure of CD4^+^ autoreactive T cell function. Such assays are robust and often already standardized and available as qualified assays in clinical laboratories. In recent years, peptide–MHC class II (pMHCII) multimers have emerged as a tool for analysis of antigen reactive, including autoreactive CD4^+^ T cells in blood or other accessible tissue sites directly *ex vivo* or after a period of *in vitro* restimulation with peptide (3–6). They consist of biotinylated MHC class II molecules with bound peptide multimerized with fluorochrome-labeled streptavidin (7). The peptide presented by the MHC class II molecule is the self- or autoantigen that is targeted by the tolerizing immunotherapy. Hence, antigen-specific T cell receptors (TCRs) on CD4^+^ T cells can be detected in flow cytometric assays to enumerate the number and, with cell surface-specific monoclonal antibodies, the phenotype of antigen-specific CD4^+^ T cells restricted to a particular MHC class II molecule.

Various protocols on the use of pMHCI and pMHCII tetramers and surface antibody staining to enumerate and phenotype unmanipulated cells directly *ex vivo* or *in vitro* peptide-stimulated T cells have been published in recent years (8–10). However, autoantigen-specific CD4^+^ T cells are rare in the circulation (generally less than 100/10^6^ CD4 T cells), the TCR is of low affinity and the pMHCII have a high off-rate (3, 11, 12), thus optimization of staining for consistent identification of TCR reactive with pMHCII is technically challenging. Several methodologies have been described to enhance detection, including staining cells with tetramers labeled with phycoerythrin (PE)-based fluorochromes followed by enrichment using PE-beads and magnetic-activated cell sorting (13). Alternative published approaches include the tyrosine kinase inhibitor dasatinib, to reduce TCR internalization and maximize TCR surface detection, and amplification of the tetramer signal using an antibody sandwich (7, 8, 14). There are also various options for the procurement or generation of multimer reagents, including specialized research laboratories, the NIH tetramer core facility, and several commercial suppliers, including MBL, ProImmune (PI), and Immudex.

In this methodological analysis, we compared several approaches to the staining of antigen-specific CD4^+^ T cells using PE-labeled tetramers directly *ex vivo* and tested reagents from two commercial suppliers and one specialized research laboratory to identify sources of variability and limitations of the assay. We optimized a pMHCII tetramer flow cytometry-based protocol to quantify and phenotype unmanipulated antigen-specific CD4^+^ T cells in the circulation of individuals to enable the visualization of cellular changes *in vivo*.

## Methods

### Patient Samples

Peripheral blood mononuclear cells (PBMC) were isolated from PB of rheumatoid arthritis (RA) patients visiting the outpatient clinic of the Princess Alexandra Hospital in Brisbane, Australia. The protocol was approved by the Metro South and UQ HRECs, and informed consent was obtained from all participants.

### pMHCII Tetramers

HLA-DRB1*0401 or HLA-DRB1*0101 hemagglutinin_306–318_ and collagen type II_259–273_ monomers were generated as previously described (14) and tetramerized using streptavidin-PE (BD Biosciences) or purchased either as monomers or tetramers from commercial suppliers (PI and MBL).

### Monoclonal Antibodies

The following monoclonal antibodies were used in this study: antihuman CD3 evolve™ 655 (eBioscience), antihuman CD3 BUV737 (BD Biosciences), antihuman CD4 BUV395 (BD Biosciences), antihuman CD4 PerCP/efluor710 (eBioscience), antihuman CD4 AlexaFluor 700 (BD Biosciences), antihuman CD11c FITC (BioLegend), antihuman CD14 FITC (BioLegend), antihuman CD16 FITC (BioLegend), and antihuman CD19 FITC (BioLegend).

### Flow Cytometric Analysis

Peripheral blood mononuclear cells were either stained directly after isolation or after storage in liquid nitrogen. Frozen PBMCs were thawed in RPMI [in the presence of 12.5 µg/ml DNase I (Sigma) until the first centrifugation step and with 6.25 µg/ml DNase I until the second centrifugation step]. Thawed cells were rested in a 37°C incubator for 15–20 min, with clumps of dead cells subsequently removed using a cell strainer (Corning). To prevent non-specific binding, FcR Blocking reagent (Miltenyi Biotech) was used according to the manufacturer’s protocol (2 µl up to 10 million cells). When a dasatinib step was included during the staining procedure, cells were incubated with 50 nM dasatinib (Selleck Chemicals) in RPMI containing 10% human AB serum, 50 U/ml IL-2 and 25 mM glucose in a 37°C waterbath for 30 min. Tetramer was then added at a concentration of 4.2 μg/ml and incubated for 1 h at 4°C. The cells were then washed once in FACS Buffer (0.1% BSA and 2 mM EDTA in PBS) and incubated with a mix of surface antibodies. Where sandwich staining was included, cells were stained with anti-PE biotin (BioLegend) at 1:1,000 or rabbit anti-PE (MyBiosource) at 1:4,000 for 20 min at 4°C after the tetramer staining. Subsequently cells were washed once in 1× FACS Buffer and incubated together with surface antibodies and streptavidin-PE (BD Biosciences) at 1:1,000 or AlexaFluor 555 goat anti-rabbit (Life Technologies) for 20 min at 4°C. To discriminate live from dead cells, cells were washed in PBS and stained using the LIVE/DEAD fixable Dead Cell Stain Kit (Invitrogen) according to the manufacturer’s protocol. All samples were acquired on a BD LSR Fortessa X20 (BD Biosciences).

## Results

### The Numbers of pMHCII Tetramer-Positive Cells Were Comparable in Frozen and Freshly Isolated Cell Samples

In clinical trials, PBMC samples are normally collected at several time points to monitor mechanism or efficacy of treatment. To internally control for experimental variation, PBMC collected from a single donor at different time points are ideally frozen, then thawed, stained and analyzed using an internally controlled, qualified assay. Collagen II_259–273_ is the dominant epitope in the murine collagen-induced arthritis model in HLA-DRB1*04:01 or HLA-DRB1*01:01 transgenic mice and the collagen II_259–273_ specific TCR repertoire in these mice is highly restricted (15). A functional T cell stimulation assay identified T cells responding to the same epitope in HLA-DRB1*04:01^+^ RA patients and healthy controls (16). Furthermore, the dominant influenza epitope HA_306–318_ can be presented by both HLA-DRB1*04:01 and HLA-DRB1*01:01 (17). HLA-DRB1*04:01-HA_306–318_ tetramers were shown to identify antigen-specific T cells in RA patients (14, 18). Thus, HA-specific CD4^+^ T cells represent an internal control for tetramer staining, independent of RA.

We first tested variability in the capacity to detect CD4^+^ antigen-specific T cells from RA patients from freshly isolated or frozen PBMC using a published pMHCII tetramer-staining protocol (7, 14). We generated tetramers specific for HLA-DRB1*04:01-collagen II_259–273_, HLA-DRB1*01:01-collagen II_259–273_, HLA-DRB1*04:01-HA_306–318_, and HLA-DRB1*01:01-HA_306–318_ and assessed antigen-specific CD4^+^ T cells in patients with an appropriate HLA type. In the first set of experiments, we endeavored to increase tetramer detection sensitivity by incubation with dasatinib and secondary antibody sandwich. Dasatinib is a tyrosine kinase inhibitor, which prevents internalization of the TCR and therefore increases the number of cell surface TCRs that can bind the tetramer and hence the detection signal. In some experiments, we included a sandwich step of biotinylated anti-PE antibody followed by streptavidin-PE or rabbit anti-PE followed by goat anti-rabbit AlexaFluor 555 to amplify the signal (7).

Fresh samples from RA patients were collected and stained immediately or frozen. Frozen cells were thawed and stained 3–8 days later with HLA-DRB1*04:01-collagen II_259–273_ or HLA-DRB1*01:01-collagen II_259–273_ tetramers. Collagen II-specific CD4^+^ T cells were detected in both fresh and frozen samples (Table 1). There was no consistent increase or decrease in tetramer frequency in frozen and thawed cells relative to freshly isolated cells from the same donor. Furthermore, in the two patients in which the sandwich step was included, the tetramer frequency was the same or increased relative to samples stained without sandwich amplification (Table 1). These results indicate that while frozen samples can be used for the detection of antigen-specific CD4^+^ T cells using pMHCII tetramers with or without sandwich amplification, the frequency of autoantigen-specific cells can vary from assay to assay in the same patient.

**Table 1:** Overview number of detected collagen II-specific CD4^+^ T cell in fresh and frozen samples.

Donor	Tetramer employed	# tetramer^+^ cells/10^6^ CD4^+^ T cells, fresh	# tetramer^+^ cells/10^6^ CD4^+^ T cells, frozen	# tetramer^+^ cells/10^6^ CD4^+^ CD4^+^ T cells, fresh^+^ sandwich	# tetramer^+^ cells/10^6^ CD4^+^ CD4^+^ T cells, frozen^+^ sandwich
1	HLA-DRB1*01:01-collagen II_259–273_	54.36	53.44		
2	HLA-DRB1*01:01-collagen II_259–273_	141.19	47.27		
3	HLA-DRB1*04:01-collagen II_259–273_	25.59	66.70	80.64	108.64
4	HLA-DRB1*04:01-collagen II_259–273_	55.82	20.05	56.72	55.81
5	HLA-DRB1*01:01-collagen II_259–273_	1.26	12.51		
6	HLA-DRB1*01:01-collagen II_259–273_	12.85	10.61		

### A Shorter Staining Process Resulted in Higher Yield of Cells for Analysis

**Figure 1:**
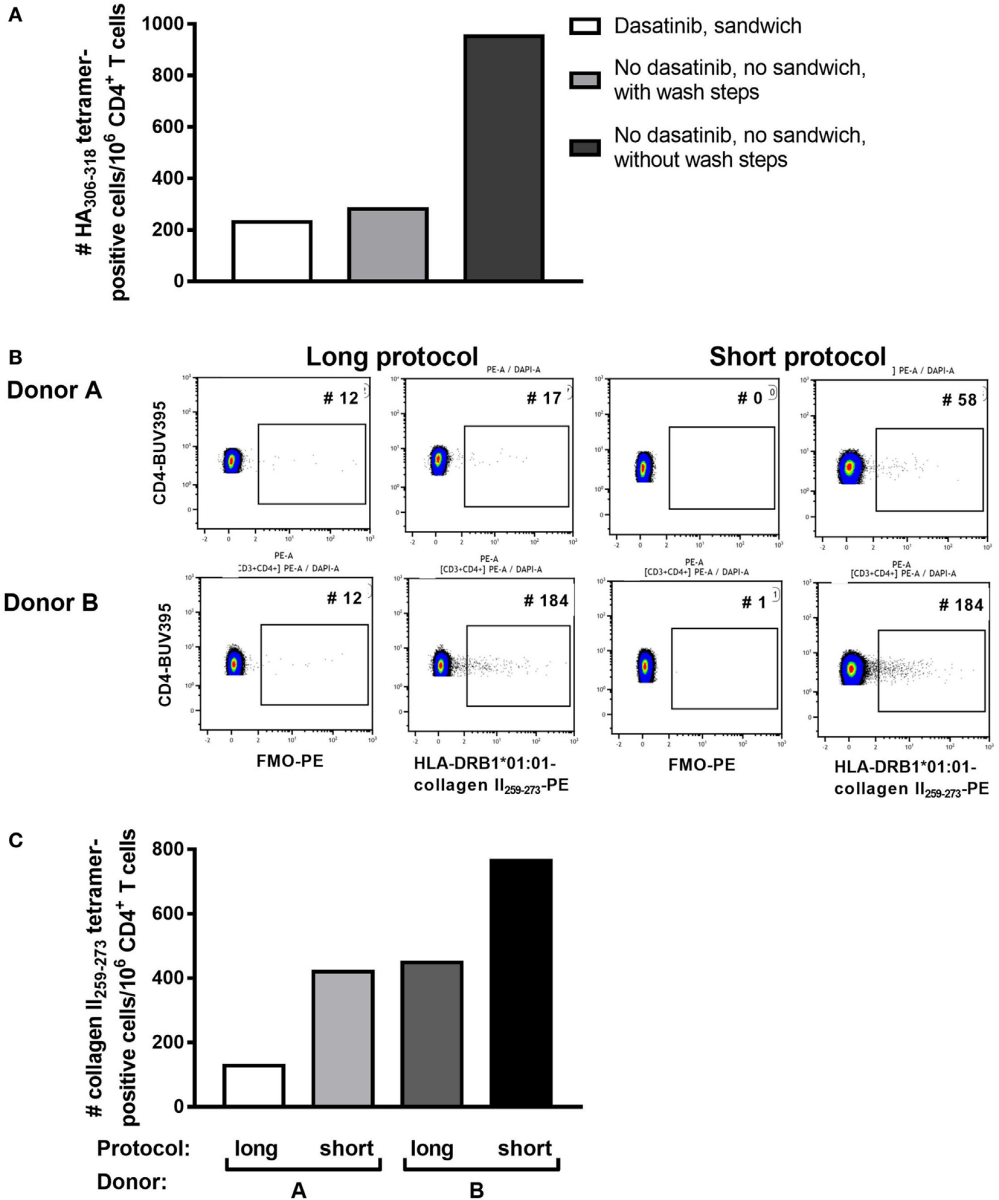
Shorter staining protocol resulted in higher numbers of tetramer-positive CD4^+^ T cells. Three staining protocols were compared as indicated. **(A)** The number of HLA-DRB1*0101 HA_306–318_-positive CD4^+^ T cells per million CD4^+^ T cells is depicted for the three protocols in peripheral blood mononuclear cells of one representative individual. **(B)** Flow cytometry plots for two patients stained with HLA-DRB1*0101-collagen II_259–273_, using either the short or the long protocol. The negative control FMO plots are shown. **(C)** The number of HLA-DRB1*0101-collagen II_259–273_^+^ CD4^+^ T cells per million CD4^+^ T cells using the short versus the long staining protocol in Donors A and B, as depicted in panel **(B)**.

PBMC, including activated PB T cells, from RA patients are fragile and have a high propensity for apoptosis in culture, which is amplified after thawing. Furthermore, we and others have shown that CD4^+^ tetramer^+^ T cells include activated memory T cells (6, 14, 18), which are particularly sensitive to manipulation *in vitro*. Given the variability in tetramer^+^ cells from assay to assay, and our observations that cell death occurred during the prolonged staining procedure required for dasatinib, sandwich amplification and multiple washes (“long protocol”), we developed an alternative protocol with minimal cell handling, i.e., short tetramer incubation step at 4°C, followed by addition of cell surface antibodies without washing in between (“short protocol”). We compared the two protocols directly in HLA-DRB1*01:01^+^ RA patients using HLA-DRB1*01:01-HA_306–318_ and HLA-DRB1*01:01-collagen II_259–273_ tetramers. The number of HLA-DRB1*01:01-HA_306–318_-specific cells was fourfold higher using the short protocol than the long protocol (Figure 1A). Where these protocols were compared head to head in two further patients, the number of HLA-DRB1*01:01-collagen II_259–273_-specific cells was twofold higher using the short protocol than the long protocol (Figures 1B,C; Table 2). We therefore compared cell yields and frequency of tetramer^+^ cells across 20 HLA-DRB1*04:01^+^ or *01:01^+^ frozen PBMC from RA donors, 10 of which were stained with the long protocol and 10 with the short protocol (Table 3). For the long protocol, the number of PBMC included per tetramer stain was 3–18 × 10^6^ cells, all of which had to be acquired on the flow cytometer to achieve sufficient frequency of tetramer^+^ CD3^+^ CD4^+^ T cells (mean 0.002%). By contrast, for the short protocol ≤5 × 10^6^ PBMC were included per tetramer stain, and of these, acquisition of 1–2 × 10^6^ cells (mean 1.42 × 10^6^, which did not use the entire tube of cells) was sufficient to achieve an improved frequency of tetramer^+^ CD3^+^ CD4^+^ T cells (mean 0.012%). Thus, the short protocol not only increased the yield of PBMC that could be acquired on the flow cytometer for analysis but also increased the efficiency of detection of tetramer^+^ cells without any increase in background staining in the FMO control (Figure 1). As a result, the number of cells required for acquisition dropped to 1–2 × 10^6^ cells, which greatly decreased acquisition time and thus reduced assay cost. These data indicate that shorter processing time without wash steps reduces loss of cells during processing, preserves viability, and increases tetramer^+^ T cell detection efficiency.

**Table 2:** Recovery and identification of tetramer^+^ cells after short and long staining protocols.

Figure	Protocol	# Cells stained × 10^6^	# Cells acquired × 10^6^	% Live CD3^+^ CD4^+^ cells	% Live tetramer^+^ CD3^+^ CD4^+^ cells	Fold increase over long protocol	# tetramer^+^/10^6^ CD3^+^ CD4^+^ cells
1A	Long	2	1.04	NA	0.0048		128
	Short + wash	2	0.82	NA	0.0076	1.6	55
	Short − wash	2	0.9	NA	0.0140	2.9	413
1B Donor A	Long	2.5	0.8	40.1	0.0051		17
	Short	2.5	0.95	24.7	0.0104	2	58
Donor B	Long	2.5	1.1	57.0	0.0256		449
	Short	2.5	1.3	58.3	0.0447	1.74	783

*In the first experiment (Figure 1A), 2 × 10^6^ peripheral blood mononuclear cells were stained per protocol, and in the second (Figure 1B) 2.5 × 10^6^ cells were stained per protocol for each of 2 donors. The percentage (%) live tetramer^+^ CD3^+^ CD4^+^ T cells identified using the long protocol was improved twofold to threefold by use of the short protocol without washes*.

**Table 3:** Identification of tetramer^+^ cells after short and long staining protocols.

HLA-DRB1*	Protocol	# Cells stained × 10^−6^	# Cells acquired × 10^−6^	% Gated live CD3^+^ CD4^+^ cells*	% Live tetramer^+^ CD3^+^ CD4^+^ cells	# tetramer^+^/10^6^ CD3^+^ CD4^+^ cells
04:01	Long	17	4.1	55.8	0.0029	52.9
04:01	Long	9	2.3	49.4	0.0019	39.8
04:01/01:01	Long	9	3.2	74.5	0.0005	7.32
04:01	Long	14	5.0	61.8	0.0038	62.5
04:01	Long	13	5.1	68.5	0.0016	23.3
04:01	Long	15	4.3	78.1	0.0013	16.4
04:01	Long	3.2	1.4	71.0	0.0056	79.5
04:01	Long	7.5	2.1	71.2	0.0024	32.7
04:01	Long	7	2.9	51.5	0.0017	33.1
Mean (SD)		10.5 (4.5)	3.4 (1.3)	64.6 (10.4)	0.0024 (0.002)	38.6 (23.0)
04:01	Short	5	1.2	71.9	0.0208	289
01:01	Short	5	1.5	77.6	0.0165	213
04:01	Short	5	1.4	71.3	0.0176	248
01:01	Short	5	2.0	72.7	0.0279	384
04:01	Short	3	1.6	70.2	0.0074	106
01:01	Short	3	1.9	76.3	0.0146	191
01:01	Short	3	1.0	51.1	0.0064	126
04:01	Short	3	1.0	63.1	0.0077	123
04:01	Short	2	1.2	75.2	0.0029	38
01:01	Short	2	1.4	51.8	0.0023	44
Mean (SD)		3.6 (1.3)	1.4 (0.3)	68.1 (9.6)	0.0124 (0.008)	176 (110)

Peripheral blood mononuclear cells from 20 rheumatoid arthritis donors were stained with collagen II_257–273_ tetramers according to HLA-DRB1* type (column 1) using the long or the short protocols. The number of cells stained and acquired as well as the percentage (%) and number (#) of tetramer^+^ T cells are shown. Whereas all cells stained were subsequently acquired for the long protocol, in the short protocol 1–2 × 10^6^ cells were collected per sample. * for the long protocol, CD3^+^ CD4^+^ T cells were gated based on exclusion of FITC^+^ lineage^−^ dump channel in combination with Aqua^+^ live/dead discriminator. For the short protocol, green live/dead discriminator was included in the FITC dump channel, which was used for exclusion.

*NA, not available*.

### Overcoming Challenges of Cell Yield after Thawing

Given that viability appears to be critical for identification of antigen-specific CD4^+^ T cells in RA PB, we compared different approaches to minimize the cell loss after thawing and to maximize the yield of cells for analysis of antigen-specific CD4^+^ T cells. First, we noted that after addition of DNase during the thawing process, free-floating DNA fragments and cell clump formation were minimized, increasing yield of cells in suspension. Second, resting thawed cells at 37°C for 20 min allowed cells to recover, and cell clumps could be removed from the cell suspension before staining, preventing clump formation during staining. We observed that clumps forming during staining are difficult to remove, often resulting in a further loss of cells. Third, as noted earlier, cells are lost with every wash step, and reducing the number of wash steps increased cell yield (Table 2; Figure 1).

### Tetramers from Different Sources Result in Staining Variability

**Figure 2:**
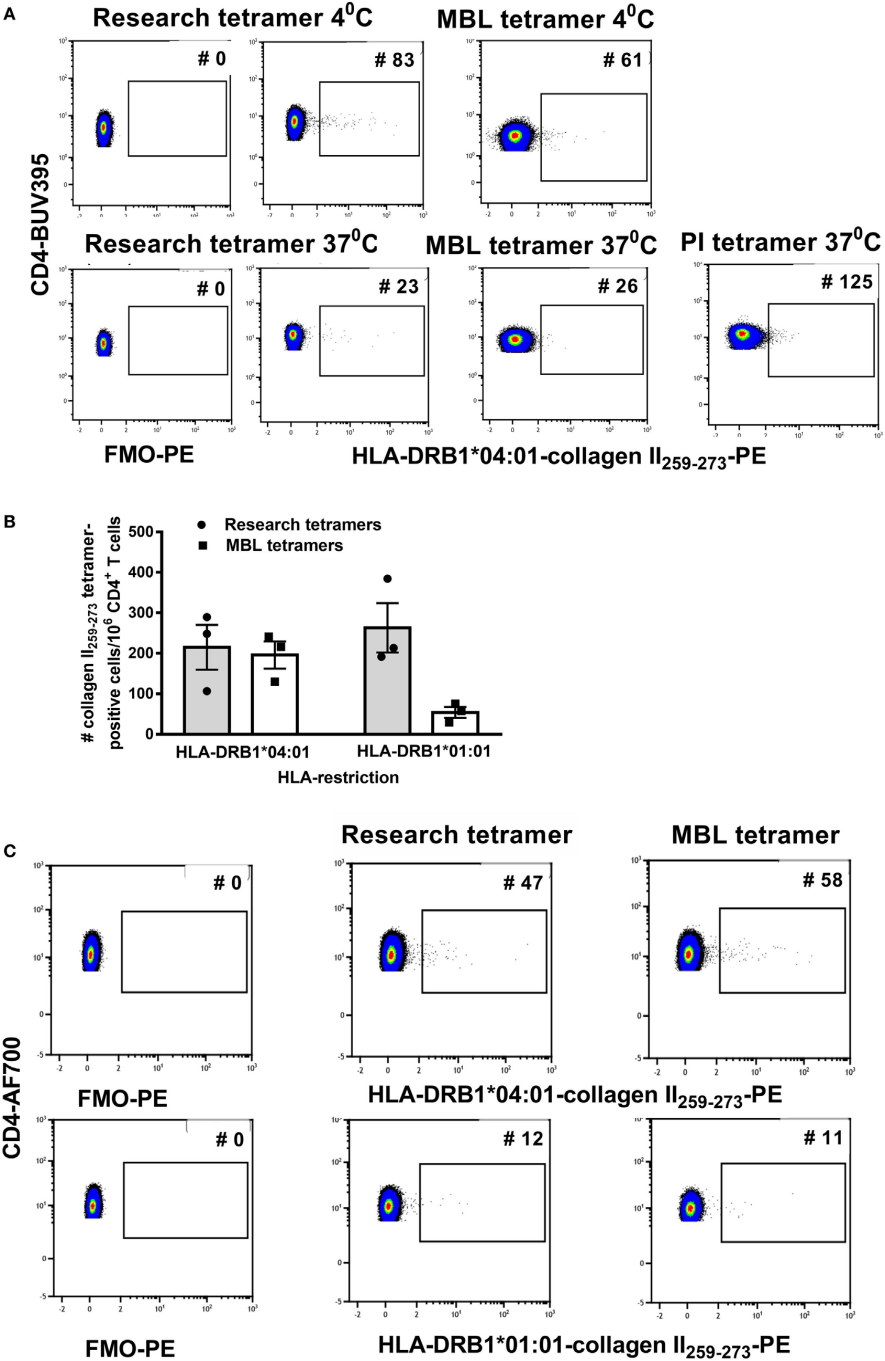
The streptavidin–biotin ratio is important for optimal tetramer formation. Research tetramers were compared with tetramers purchased from MBL and ProImmune (PI). **(A)** Flow cytometry plots of a representative donor depicting FMO-PE and staining with HLA-DRB1*0401-collagen II_259–273_ tetramers using the short staining protocol (representative of three HLA-DRB1*04:01^+^ and three HLA-DRB1*01:01^+^ donors with rheumatoid arthritis). **(B)** The number of tetramer-positive cells per million CD4^+^ T cells identified using HLA-DRB1*0401-collagen II_259–273_ and HLA-DRB1*0101-collagen II_259–273_ tetramers from two sources (*n* = 3 replicates per donor), staining at 4°C. **(C)** Flow cytometry dot plots depicting the number of tetramer-positive cells using tetramers generated from monomers as depicted, from two sources using the same formula to calculate the streptavidin–biotin ratio and each stained at a concentration of 4.2 µg/ml (representative of three individual donors). In panels **(A,B)**, staining with research and MBL tetramers was carried out at 4 and 37°C and with PI tetramers at 37°C. In panel **(C)**, all tetramers were stained at 4°C.

For a clinical trial, monomers or tetramers qualified by a commercial supplier are preferred to research reagents. To test variability between reagents we compared staining with custom HLA-DRB1*04:01-collagen II_259–273_ and HLA-DRB1*01:01-collagen II_259–273_ tetramers purchased from two commercial suppliers, MBL and PI, with research tetramers produced as previously described (14). Cells were stained according to the manufacturer’s protocol, which was very similar to the short protocol described earlier except that PI and MBL recommended staining cells with tetramer for 2 h at 37°C. Since the above tests using research tetramers were optimized at 4°C, we compared the manufacturer’s protocol staining cells with tetramers for 2 h at 37°C with our protocol staining cells with tetramers at 4°C for 1 h. Compared with the FMO and research tetramers, the commercial tetramers had an overall spreading of the CD3^+^ CD4^+^ tetramer-negative PE signal and a reduction in fluorescence intensity of the cloud of HLA-DRB1*04:01-collagen II_259–273_^+^ T cells (Figure 2A). Staining at 37°C increased cell clumping and reduced tetramer staining using either research or MBL tetramers. HLA-DRB1*01:01-collagen II_259–273_ T cells were virtually undetectable when staining with MBL tetramers (Figure 2B).

Commercially supplied tetramers have been tetramerized from biotinylated monomers using undisclosed methodology and reagents, and a volume/test rather than concentration is provided by the manufacturer. The observed difference in PE signal between research and commercial tetramers suggested differences in staining may be related to the streptavidin–biotin ratio used for tetramerization and/or tetramer concentration when staining. To be able to standardize across comparisons, we purchased DRB1*04:01-collagen II_259–273_ and HLA-DRB1*01:01-collagen II_259–273_ biotinylated monomers from MBL, requested details of % biotinylation and monomer concentration and tetramerized each, based on the percentage of monomer biotinylation according to the formula in Table 4. The resulting tetramers generated in our laboratory from MBL or research biotinylated monomers gave comparable staining on the same patient cell samples (Figure 2C) when stained at 4.2 µg/ml at 4°C. These results indicate that for optimal reproducibility between tetramer production facilities and laboratories receiving different batches of tetramers, it is important to standardize the procedures for tetramerization from biotinylated monomers, maintaining a consistent source of streptavidin fluorochrome, a consistent streptavidin–biotin ratio, and a consistent tetramer concentration in the staining reaction.

**Table 4:** Formula for streptavidin-PE volume calculation for tetramerization of biotinylated monomers.

Volume Strep-PE to add 10 times (μl) = ((amount monomer (μg) × 0.74285714285714)/8)/5
Volume monomer needed = amount monomer (μg)/concentration biotin (mg/ml)
Concentration biotin = concentration monomer × percentage biotinylation

*The rationale is as follows: Mass of biotinylated monomer used/70,000 (M_w_ of Class II HLA-DR) = Volume of Strep-PE/52,000 (M_w_ of Strep-PE) × 8 (1:8 ratio of Strep-PE to biotinylated monomer needed) × 0.5 (concentration Strep-PE in mg/ml)*.

### Optimizing Fluorochromes and Gating Strategy

**Figure 3:**
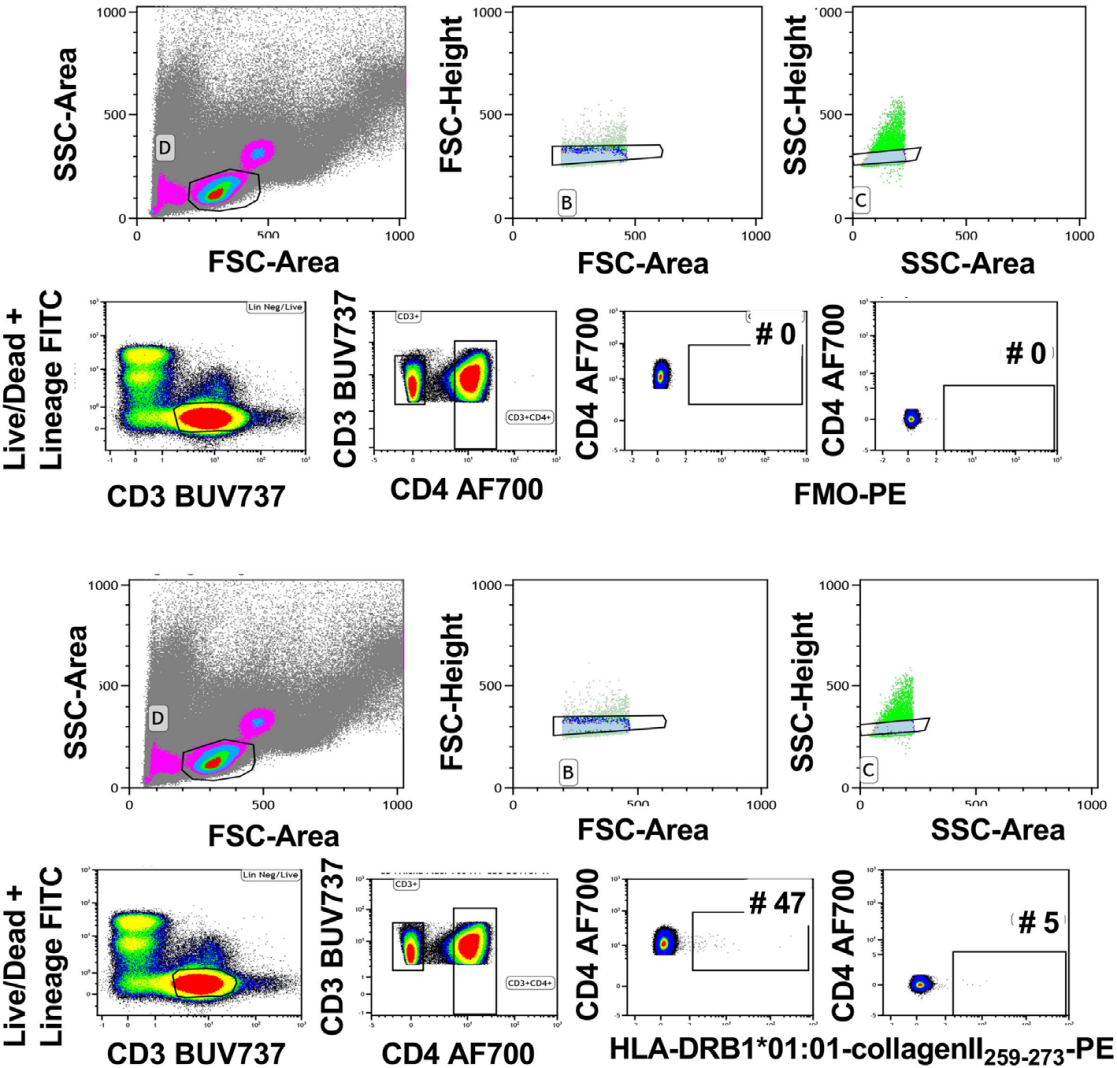
Gating strategy for tetramer analysis. Live cells were gated using forward and side scatter. Subsequently, single cells were gated using forward scatter height and area followed by side scatter height and area (upper panels). Live CD3^+^ cells were gated using Lineage/LIVE/DEAD marker negative and CD3-positive cells. Next, CD4^+^ T cells were gated by gating on the CD3^+^ CD4^+^ double-positive cells. The FMO sample was used to determine the tetramer-positive gate. The gate was set on the CD4-postive cells that are negative in the tetramer channel (middle panels). This gate was used to identify the CD4^+^ tetramer^+^ cells in the rest of the samples (bottom panels). Representative of 40 experiments.

Tetramer intensity varies with the tetramerization procedure (Figure 2A), and streptavidin-PE intensity varies somewhat depending on the manufacturer (data not shown). Therefore, when comparisons are required, it is important to standardize these parameters for the entire study and for all laboratories involved. Furthermore, after construction from monomers, tetramers will lose integrity after 2–3 months. Therefore, it is important to calculate the assay time required for a batch of clinical samples so that they can be analyzed with a single monomer batch. The brightest signal relative to background will be obtained with streptavidin conjugated to fluorochromes such as PE, APC, or Brilliant Violet (BV) 421. We have successfully combined tetramers of different pMHC specificities conjugated to BV421 and PE, respectively to identify single or double labeling, i.e., to determine TCR cross-reactivity (6). Reduction in myeloid cell background is avoided with the use of Fc block, live/dead and lineage exclusion gating, and optimization of CD3 and CD4 staining to ensure tight T cell gating, with, minimal background staining of CD3^+^ CD4^−^ T cells. Figure 3 is an example of the gating strategy. For cell surface staining, we avoid the use of tandem dyes based on PE, which could degrade and give false positive PE signal in the tetramer-PE channel. Staining and tetramerization protocols are provided in Supplementary Material.

## Discussion

As a result of advances in antigen-specific immunotherapeutic approaches to induce tolerance, such as the delivery of antigen-exposed tolerogenic dendritic cells or other forms of antigen delivery designed to promote tolerance, early-stage translation to clinical trials in autoimmune disease has begun (1, 19–22). While animal data are promising, it is likely that for consistent demonstration of robust clinical outcomes from antigen-specific approaches, ongoing basic and clinical development from a number of angles will be required. During the current exploratory phase in this field, it is essential to gather as much information as possible on the CD4^+^ T cells responding to delivered antigen to assess and to improve on the outcomes of clinical trials of antigen-specific immunomodulatory approaches. pMHCII multimers represent an excellent tool to monitor antigen-specific CD4^+^ T cell responses, as they can be used to quantify the frequency and assess the phenotype of antigen-specific T cells (6, 14). In addition, as staining reagents, they can facilitate in-depth, exploratory single cell analyses, such as TCR sequencing, transcriptomics, assessment of oligoclonal expansion, and cloning (6, 23). Furthermore, because they can be applied to flow cytometry or cell sorting, multimers are much more sensitive and versatile than *ex vivo* analyses such as ELISPOT, which struggle to detect low level cytokine responses above background produced by autoantigen-specific memory T cells (24–26).

However, due to the low avidity and high off-rate between pMHCII and the TCR, these assays are technically challenging for use in clinical trials. Here, we investigated reagent and assay limitations that could impact the use of peptide–HLA–DR tetramers in clinical trial settings for immunomonitoring. We describe a peptide–MHC class II flow cytometry-based assay to quantify and phenotype antigen-specific CD4^+^ T cells. We show that this assay can be used in frozen PBMC, but that optimal cell yield for identification of antigen-specific CD4^+^ T cells after thawing is best achieved by reducing staining time before analysis on the flow cytometer. Elimination of dasatinib and sandwich amplification steps, combining staining steps, reducing the number of washes, adding DNAse during thawing, and resting of the cells after thawing all improved efficiency of identification of tetramer^+^ CD4^+^ T cells. As a result of these modifications, replicates of the same tetramer stain on the same sample showed good reproducibility of cell frequency (Figure 2B). Furthermore, to optimize the number of tetramer-positive cells detected, the source of monomers and the tetramerization formula must also be taken into account, as this will affect the staining outcome. When tetramerized similarly from biotinylated monomers and stained at the same concentration, replicates of the same samples stained with tetramers from different sources also showed good reproducibility. Furthermore, we provide an example of gating strategy and tips on fluorochrome use. Previously we have demonstrated by single cell sorting using pMHCII multimers that HLA-DRB1*14:02-Vimentin_59–71_^+^ and HLA-DRB1*14:02-Vimentin_59–71_Cit64^+^ autoreactive T cells are oligoclonally expanded in the blood of RA patients, and that a representative HLA-DRB1*14:02-Vimentin_59–71_-reactive TCR cloned from the identified sequences could be restimulated with HLA-DRB1*14:02-Vimentin_59–71_ or HLA-DRB1*14:02-Vimentin_59–71_Cit64, but not HLA-DRB1*14:02-CLIP (6). These studies indicate that autoantigen-specific cells can be identified, cloned and characterized for peptide specificity and cross-reactivity using the methods described here for pMHCII staining.

Even though the described modifications resulted in reproducible identification of self-antigen-specific CD4^+^ T cells, there are limitations to tetramer assays apart from the low cell frequencies identified by staining. Due to the low avidity between self-antigen–MHCII complexes and TCRs, the mean fluorescence intensity of tetramer staining is low. As a result background and positive staining tend to merge—hence attempts to amplify staining signal with dasatinib and sandwich stains—which creates difficulty in setting the cutoff for positive staining. This is much less of a problem for tetramers of higher avidity, e.g., pMHCI tetramers, HLA–DQ–gliadin peptide tetramers, or tetramers for B cell receptor staining. To determine the cutoff between background and positive staining of autoantigen-specific CD4 tetramers, we have used FMO gating. This is because control peptide tetramers typically identify T cells with low-avidity staining to the control peptide and mismatched HLA tetramers typically identify low numbers of alloreactive T cells. While it is useful to compare frequency of T cells with these reactivities relative to the antigen-specific T cells of interest, these controls are unhelpful for discriminating between background and positive staining. An alternate strategy is to check background tetramer staining in the CD3^+^CD4^−^ T cell gate. Using the strategies outlined here, this was demonstrated to be minimal (Figure 3). However, it is important that the antigen-specificity of tetramer^+^ T cells be verified by other means, i.e., transduction of paired alpha-beta TCR derived from tetramer^+^ T cells and restimulation with the appropriate antigen presenting cells and peptides. Finally, general availability of a range of positive control reagents (antigen-specific, HLA-restricted CD4 T cell clones or hybridomas) would be helpful to validate tetramers produced by research labs and facilities, as well as those sold by companies. Although some of these autoantigen-specific reagents exist, these have been challenging to produce on a broad scale so far.

Since the development of technologies to produce pMHCII tetramers, methodologies for effective, specific and reproducible identification of antigen-specific CD4^+^ T cells from blood and other tissues of mice and humans have been improving. While these methods represent our current optimized techniques for reproducibility in clinical studies, we anticipate that research methods will continue to improve with advances in reagents and analytical technologies, such as mass cytometry. This evolution will likely be rapid due to a strong drive to understand mechanisms and responses to antigens in autoimmune diseases and the outcomes of immunotherapies designed to modify those responses.

## Author Contributions

Contributed and/or interpreted data: DJ, NR, and HN. Provided reagents: JR, KL and HR. Wrote paper and study design: DJ, NR, and RT. All the authors read and approved the final manuscript.

## Conflict of Interest Statement

RT holds and has filed patents surrounding technology for targeting DCs for antigen-specific tolerance and is a director of the spin-off company, Dendright, which is commercializing antigen-specific immunotherapy in collaboration with Janssen Biotech Inc. All other authors declare that the research was conducted in the absence of any commercial or financial relationships that could be construed as a potential conflict of interest.
