# Supplementary material for: Flow Cytometric Clinical Immunomonitoring Using Peptide–MHC Class II Tetramers: Optimization of Methods and Protocol Development
Source: Front Immunol. 2018 Jan 22;9:8. doi: 10.3389/fimmu.2018.00008 (PMC5786526; doi:10.3389/fimmu.2018.00008)
Supplement: Supplementary file 1 [file Presentation_1.PDF]

SUPPLEMENTARY METHODS

Tetramer staining protocol

1. Thaw PBMCs including 12.5 μg/ml DNAse in the first centrifugation step and 6.25 μg/ml DNAse in the second centrifugation step.
2. Let the cells rest at 37^{0}C for 15-20 minutes.
3. Remove the dead cell clumps using a cell strainer.
4. Block the Fc receptors by adding 2 μl/ml Fc Blocking reagent. Up to 10 million cells/ml. Incubate for 10 minutes at 4^{0}C.
5. Incubate the cells with tetramer at 4.2 μg/ml for 1 hour at 4^{0}C. The optimal tetramer concentration can be determined using a titration curve or per manufacturer's recommendation.. Include 2 million PBMC per tetramer stain and always include a FMO sample. Use 1 million PBMC for the FMO sample, which will not be stained with the tetramer, but the surface marker staining will be included.
6. Add in the surface marker antibodies and incubate for 20 minutes at 4^{0}C. Avoid adding surface markers that are coupled to PE tandem dyes to avoid false-positive signal in the PE channel. Surface markers should include lineage exclusion markers. In the examples shown, FITC-labeled CD8, CD14, CD16, CD19, CD11c and CD56 were used. Preferably, the PE signal is detected using a yellow/green laser in the flow cytometer instead of a blue laser, to decrease spectral overlap.
7. Wash the cells with saline.
8. Add LIVE/DEAD cell Fixable Green discriminator stain in saline for 10 minutes at 4^{0}C.
9. Wash cells with FACS buffer.
10. Resuspend cells in 200 μl FACS buffer for acquisition on the same day. Acquire at least 1 million cells per sample stained with tetramer.
11. Gating steps:
    a. Gate on live cells using forward and side scatter.
    b. Gate on single cells using forward scatter height and area followed by side scatter height and area.
    c. Gate on the live CD3+ cells by gating on the lineage/LIVE/DEAD marker negative and CD3 positive cells.
    d. Gate on the CD4+ CD3+ double positive cells to identify the CD4+ T cells.
    e. Use the FMO sample to determine the tetramer-positive gate. Plot CD4 against the tetramer channel and set the gate on the CD4-positive cells that are negative in the tetramer channel.
    f. Use this gate to identify the CD4+ tetramer+ cells in the rest of the samples.

Tetramerisation protocol

1. Use the formula in Table 3 to calculate the volume of streptavidin-PE to be added. The amount of biotinylated monomer to be used (μg) depends on the quantity of tetramer to be made.
2. Add the indicated volume of biotinylated monomers to a 1.5-ml Eppendorf tube and add the indicated volume of streptavidin-PE to the tube. Incubate for 10 minutes in the dark at room temperature.

3. Repeat addition of streptavidin-PE with the 10 minutes incubation, 9 times (total of 10 times).
4. Once completed, keep the tetramers at 4^0C protected from light. The tetramers will be stable for 3 months. Do not freeze the tetramers.
